# Using Artificial Intelligence With Natural Language Processing to Combine Electronic Health Record’s Structured and Free Text Data to Identify Nonvalvular Atrial Fibrillation to Decrease Strokes and Death: Evaluation and Case-Control Study

**DOI:** 10.2196/28946

**Published:** 2021-11-09

**Authors:** Peter L Elkin, Sarah Mullin, Jack Mardekian, Christopher Crowner, Sylvester Sakilay, Shyamashree Sinha, Gary Brady, Marcia Wright, Kimberly Nolen, JoAnn Trainer, Ross Koppel, Daniel Schlegel, Sashank Kaushik, Jane Zhao, Buer Song, Edwin Anand

**Affiliations:** 1 Department of Biomedical Informatics University at Buffalo Buffalo, NY United States; 2 Bioinformatics Laboratory Department of Veterans Affairs VA Western New York Healthcare System Buffalo, NY United States; 3 School of Engineering University of Southern Denmark Odense Denmark; 4 Pfizer, Inc. New York, NY United States

**Keywords:** afib, atrial fibrillation, artificial intelligence, NVAF, natural language processing, stroke risk, bleed risk, CHA2DS2-VASc, HAS-BLED, bio-surveillance

## Abstract

**Background:**

Nonvalvular atrial fibrillation (NVAF) affects almost 6 million Americans and is a major contributor to stroke but is significantly undiagnosed and undertreated despite explicit guidelines for oral anticoagulation.

**Objective:**

The aim of this study is to investigate whether the use of semisupervised natural language processing (NLP) of electronic health record’s (EHR) free-text information combined with structured EHR data improves NVAF discovery and treatment and perhaps offers a method to prevent thousands of deaths and save billions of dollars.

**Methods:**

We abstracted 96,681 participants from the University of Buffalo faculty practice’s EHR. NLP was used to index the notes and compare the ability to identify NVAF, congestive heart failure, hypertension, age ≥75 years, diabetes mellitus, stroke or transient ischemic attack, vascular disease, age 65 to 74 years, sex category (CHA_2_DS_2_-VASc), and Hypertension, Abnormal liver/renal function, Stroke history, Bleeding history or predisposition, Labile INR, Elderly, Drug/alcohol usage (HAS-BLED) scores using unstructured data (International Classification of Diseases codes) versus structured and unstructured data from clinical notes. In addition, we analyzed data from 63,296,120 participants in the Optum and Truven databases to determine the NVAF frequency, rates of CHA_2_DS_2_‑VASc ≥2, and no contraindications to oral anticoagulants, rates of stroke and death in the untreated population, and first year’s costs after stroke.

**Results:**

The structured-plus-unstructured method would have identified 3,976,056 additional true NVAF cases (*P*<.001) and improved sensitivity for CHA_2_DS_2_-VASc and HAS-BLED scores compared with the structured data alone (*P*=.002 and *P*<.001, respectively), causing a 32.1% improvement. For the United States, this method would prevent an estimated 176,537 strokes, save 10,575 lives, and save >US $13.5 billion.

**Conclusions:**

Artificial intelligence–informed bio-surveillance combining NLP of free-text information with structured EHR data improves data completeness, prevents thousands of strokes, and saves lives and funds. This method is applicable to many disorders with profound public health consequences.

## Introduction

### Background

Atrial fibrillation (AF), the most common type of arrhythmia [1,2], consists of nonvalvular AF (NVAF) and valvular AF (VAF) [1]. NVAF comprises approximately 70% of AF and currently affects approximately 5.8 million US patients and approximately 11 million in Europe on VAF results in a five times greater risk of stroke [3] and causes approximately 15% of all strokes [2,4]. Anticoagulation treatment dramatically reduces one’s odds of a stroke to <0.5% on average.

The incidence of stroke with AF has prompted the development of scoring risk systems to guide anticoagulation treatment [5,6]. In 2014, the American Heart Association, American College of Cardiology, and Heart Rhythm Society advocated for AF practice guidelines via the use of congestive heart failure, hypertension, age ≥ 75 years, diabetes mellitus, stroke or transient ischemic attack, vascular disease, age 65 to 74 years, sex category (CHA_2_DS_2_‑VASc) scores that combine the CHADS_2_ score with additional moderate risk factors [2,7]. Individuals’ stroke risks should inform therapeutic options, which may include anticoagulants [7]. The Hypertension, Abnormal liver/renal function, Stroke history, Bleeding history or predisposition, Labile INR, Elderly, Drug/alcohol usage (HAS‑BLED) score is a practical tool to assess individuals’ risk of major bleeding and to guide anticoagulant therapy [8,9]. Researchers posit that the assessment of bleeding risk factors—age, uncontrolled hypertension, ischemic heart disease, and prior ischemic stroke—may improve individualized treatment for AF.

However, despite strong recommendations, oral anticoagulation (OAC) for NVAF patients remains low, with rates ranging from 39%-65% [10]. Disease surveillance and clinical decision support could help detect potential candidates who could benefit from this therapy. Automatic extraction from electronic health records (EHRs) has been shown to aid health care providers by making health care information easily accessible and helping with risk calculation [11,12]. Using these tools could reduce clinicians’ computer time for data retrieval and data entry and could facilitate capturing all qualifying patients [13].

### The Need for Natural Language Processing

Although EHRs contain an abundance of codified information, factors related to the assessment of NVAF are often poorly reflected in structured data [11]. Clinical text harboring rich contextual medical information is unstructured and in free-text form. Extracting information from a clinical text remains challenging because of context-specific abbreviations, refusal to adhere to typical language conventions, and because text often includes a broad range of specific medical terms. To retrieve information from a clinical text, multiple natural language processing (NLP) approaches have been developed, including those that extract clinical entities and map them to clinical terminologies such as SNOMED CT (Systematized Nomenclature of Medicine–Clinical Terms) [14].

To capture all potential patients with NVAF and of CHA_2_DS_2_‑VASc >1 who would benefit from appropriate anticoagulation therapy, we developed a method to automate risk scoring systems using a combination of multiple EHR data sources for diagnostic information, namely the International Classification of Disease (ICD) codes and clinical notes and lists. As natural language processors are expensive to develop and require individual tuning for each task or disease area, we make use of a high definition-NLP (HD-NLP) method that uses semisupervised learning to surpass the classification performance that could be obtained either by discarding the unlabeled data and performing supervised learning or by discarding the labels and performing unsupervised learning [15]. We compare the advantages of using NLP tools for NVAF phenotyping and calculate the risk scores of using structured ICD data alone.

## Methods

This study compares the effectiveness of identifying NVAF patients using three methods: (1) structured EHR data, (2) a combination of structured EHR data and NLP-analyzed existing free text (EHR notes, problem lists, and laboratories), and (3) clinicians’ assessments of NVAF patients (*the gold standard*). We used NLP of the EHRs’ free text to improve the identification of NVAF patients and to assess their stroke and bleeding risks more accurately. We verified the improvement in the identification of NVAF cases and in determining the CHA_2_DS_2_-VASc and HAS-BLED scores. We then examined the rates of NVAF and treatment in patients with a CHA_2_DS_2_-VASc of ≥2 and no contraindications to treatment to determine the results from our local population. Finally, we extrapolated our findings on NVAF numbers to the US population and disease costs.

### Study Populations

We had two samples: a local Western New York population of 96,681 individuals and 63,296,120 participants from the Optum and Truven databases.

#### Sample 1: Local

To understand the effectiveness of the system in identifying NVAF patients who should be treated and are not currently on OAC therapy, we abstracted a set of 96,681 participants (aged 18-90 years) from the Allscripts outpatient electronic records at the University at Buffalo’s (UBMD) faculty practice. The research was approved by the institutional review board of the University of Buffalo.

Patient data were abstracted from 2010 to September 21, 2015, before the switch to ICD-10, allowing consistent use of ICD-9 terminology and sufficient follow-up data for the study period. This yielded 212,343 patients. Of those 212,343 patients, 96,681 (45.53%) had notes and were seen for ≥1 outpatient visits (Multimedia Appendix 1, Figure S1). Outcomes from these data included rates of AF, NVAF, and VAF diagnosis, components of the CHA_2_DS_2_-VASc and HAS-BLED scores, relevant contraindications, OAC treatment, and demographic variables. We excluded patients if they were on oral antithrombotic therapy for indications other than NVAF, had a mechanical prosthetic valve, had a hemodynamically significant mitral stenosis or significant aortic stenosis, were pregnant, had a transient AF because of reversible conditions, or had active infective endocarditis (Multimedia Appendix 1, Figure S2). We developed the NVAF cohort using ICD-9 codes (structured data) and ICD-9 and NLP (structured-plus-unstructured) of EHR notes and patient problems. AF and atrial flutter were defined by ICD-9 codes 427.31 and 427.32 and by SNOMED CT codes 49436004 and 5370000 with all subtypes in the hierarchy.

The structured data–only method used ICD 9 codes from problem lists, medications, and demographics. The structured-plus-unstructured method added clinical notes, vital signs, laboratory findings, and text from the problem list using HD-NLP for codification [14,16-18]. Free text elements were coded using SNOMED CT, a general description logic–based nomenclature of clinical medicine. Specific code inclusions can be found in Multimedia Appendix 1, Figure S3.

We then compared the accuracy of structured data alone with the structured-plus-unstructured EHR data derived using the HD-NLP system, focusing on the two models’ abilities to identify true cases of NVAF and to determine stroke and bleeding risks (CHA_2_DS_2_-VASc and HAS-BLED scores).

#### Subsample of the Local Data

For validation of the accuracy of NLP, we used a gold standard created by human review (BS, JZ, EA, and SS) from a random sample of 300 patients. To verify the NVAF identification and CHA_2_DS_2_-VASc and HAS-BLED scores, we used this 300-patient random sample from our NVAF patients, which were dual human reviewed. We also looked to determine how much better structured-plus-unstructured data were in the identification of NVAF cases and in the determination of the CHA_2_DS_2_-VASc and HAS-BLED scores.

The human review data set was independently examined by 4 clinicians, each performing 150 reviews on deidentified patient encounters from the EHR. Each clinician made a judgment as to whether the patients had sustained NVAF and whether the patient had each of the components of the CHA_2_DS_2_‑VASc and HAS-BLED scores. If there were disagreements, a fifth clinician adjudicated.

Calculations determined that 300 patients were needed for 90% power to predict a 5% change in accuracy given a two-sided alpha of .05, assuming a standard accuracy of 73% based on ICD-9 codes [19]. Multimedia Appendix 1, Figure S1 presents the decision tree and sample numbers, and Multimedia Appendix 1, Figure S2 illustrates the randomization scheme.

#### Sample 2: National—Optum and Truven Databases

We analyzed the claims data from 63,296,120 participants in the Optum and Truven databases from October 2015 to September 2016 to determine the frequency of NVAF, rates of CHA_2_DS_2_‑VASc ≥2, and no contraindications to OAC, rates of stroke and death in the untreated NVAF, strokes and death in the large claims database, and the first year’s cost after stroke [20,21]. Cost differences were based on 1-year cost before and after the stroke, adjusted for inflation.

We then extrapolated our findings to the US population.

### Findings for NLP

We made use of an HD-NLP to rapidly assign ontological terms to the text in patient records (Multimedia Appendix 1, Figure S5) [14,16,17]. HD-NLP is a full-function NLP processing pipeline that takes sentences, parses them by their parts of speech, and builds a full semantic parse in memory; then, an ontological coder works by matching words to ontology terms, with the longest match being preferred. We used basic formal ontology as an upper-level ontology to index the data from individual trials [18]. We also used the ontology of biomedical investigation and SNOMED CT as our main ontologies [22,23].

A level of syntactic processing was required to match text with ontological terms. The linguistic representation is specified in language models. Of primary concern here was an English language model to identify sentences, phrases, words, and parts of speech. Terms from the input ontologies were then assigned to spans of text. String matching techniques allowed for inexact matches influenced by the underlying language model. The structures of the free‑text medical records were captured and stored.

To develop the NVAF model, we used a semisupervised learning algorithm training set with 36,268 patients from the Allscripts EHR UBMD faculty practice data from 2007 to 2008, with 1972 AF cases and 1795 NVAF cases to determine the best SNOMED CT codes to match the case definition. As most clinical texts are unlabeled, semisupervised learning leverages a small amount of labeled data with a large amount of unlabeled data. Researchers have shown that large amounts of unlabeled data, when used in conjunction with a limited amount of labeled data, can produce considerable improvement in learning accuracy, especially with assistance from subject matter expert’s annotation of the training set’s false positive and false negative results from each training iteration [14]. All cases were coded using HD-NLP with SNOMED CT codes (the unsupervised portion of the study). Where the SNOMED CT codes and ICD-9 codes agreed that the patient had NVAF, we called that a true positive case. The same logic was used to determine true negatives. Where either coding system disagreed, our clinician (PE) reviewed the case and decided. After reviewing the false positive and false negative cases from the training data set, we used additional synonymy to the terminology and selected a more appropriate set of codes for each rule in the definition. This process was iterated on the training set until we met our accuracy goals.

### Statistical Analysis

Statistical analyses were conducted using R 3.3.2. A random gold standard sample of 300 patients was taken from the sample 1 AF cohort defined by both ICD and HD-NLP. Interrater agreement was assessed using the two-way random effects model for intraclass correlation coefficient, with two-sided 10,000 samples bootstrapped 95% CI, treating the risk scores as continuous. Cohen κ with two-sided 10,000 samples bootstrapped 95% CI assessed the interrater reliability of each individual component of the scores, NVAF and AF.

The accuracy of the structured data alone was compared with structured-plus-unstructured data for the outcomes of NVAF, CHA_2_DS_2_-VASc score, and HAS-BLED score in the random sample. Cohen κ with two-sided bootstrapped CIs was calculated as a measure of reliability between the gold standard and the structured and structured-plus-unstructured data. For sensitivity and specificity, a hypothesis test comparing structured with structured-plus-unstructured data was assessed using either the McNemar test for paired observations or the binomial exact test. For positive and negative predictive values, a generalized score statistic proposed by Leisenring et al [19] was used for comparison.

As the CHA_2_DS_2_-VASc and HAS-BLED scores are on ordinal scales from 0 to 9, we analyzed the area under the receiver operator characteristic curve (ROC) using the C-Index and Somer D, based on ordinal logistic regression, where probabilities were modelled as *P(Y≥k|X)*, where k defines the cut-offs from 0 to 9 that the score can take. We hypothesized that the structured and NLP data were more concordant than the structured-only data compared with the gold standard between the ordinal gold standard score and the ordinal method score.

We contrasted our findings with the clinical judgments from the physician review of the 300 patients, categorized as contraindicated (Multimedia Appendix 1, Table S1) or not on OAC, would or would not benefit from OAC, and not on OAC. To determine the potential effects of adopting the NLP-enabled method with structured-plus-unstructured data, the accuracy data of the structured and NLP data method were used to extrapolate the findings for all untreated US patients in the Optum and Truven data sets with no contraindications to OACs. Then, the potential savings from reduced strokes were derived and compared with the prevailing structured-only method.

## Results

### NLP Results

From the Allscripts UBMD practice EHR data, we found 2722 potential patients with NVAF using the structured and NLP method and 1849 cases using only ICD-9 codes. The use of NLP by combining structured-plus-unstructured data improved sensitivity by 32.1%, that is, 873/2722 (*P*<.001) in determining the NVAF population. In the random sample, participants were on average 72 years old (mean 72.7, SD 13.6), 41.3% (125/300) were female, and 86.3% (259/300) were White. The true NVAF population within the random sample, as determined by clinician review, was 88% (264/300) of cases with an average age of 73 (mean 73.4, SD 13.0), of which 41.7% (110/264) were female, and 87.1% (230/264) were White. The assessment of agreement between clinicians and interrater reliability was high for the CHA_2_DS_2_-VASc score (odds ratio [OR] 0.796, 95% CI 0.725-0.853 and OR 0.878, 95% CI 0.838-0.909) and adequate for the HAS-BLED score (OR 0.609, 95% CI 0.51-0.692 and OR 0.675, 95% CI 0.544-0.77). Cohen κ, depending on whether an outcome was a rare event, ranged from –0.080 to 0.84.

When we tested this in the human review of the 300 cases, we found a 46% improvement in sensitivity (Table 1), which is greater than the 32.1% improvement seen with the automated method.

**Table 1:** Clinician review (gold standard): comparison of outcomes for structured and structured-plus-unstructured data against the gold standard for identifying a case as nonvalvular atrial fibrillation.

Outcome	Structured surveillance	Structured and NLP^a^ surveillance	*P* value
Sensitivity, OR^b^ (95% CI)	0.54 (0.48-0.60)	1 (0.979-1)	<.001
PPV^c^, OR (95% CI)	0.95 (0.90-0.98)	0.93 (0.893-0.956)	.24
F^d^ score	0.686	0.964	N/A^e^

^a^NLP: natural language processing.

^b^OR: odds ratio.

^c^PPV: positive predictive value.

^d^For case finding of nonvalvular atrial fibrillation.

^e^N/A: not applicable.

Thus, the structured-plus-unstructured surveillance showed that the sensitivity for CHA_2_DS_2_-VASc ≥2 and HAS-BLED≥3 scores was significantly better than that for structured data alone (*P*=.002 and *P*<.001, respectively). The specificities of the two methods were not statistically different for CHA_2_DS_2_-VASc and favored the structured method for HAS-BLED (Table 2). The positive predictive value (PPV; precision) also improved for the HAS-BLED score using the structured-plus-unstructured method (Table 2) but was not statistically different from the structured data for the CHA_2_DS_2_-VASc score. However, the negative predictive value improved for both scores using the structured-plus-unstructured method. No cases identified by the structured method were missed by the structured-plus-unstructured method.

**Table 2:** Comparison of outcomes for structured and structured-plus-unstructured surveillance against the clinician review (gold standard) for identifying Hypertension, Abnormal liver/renal function, Stroke history, Bleeding history or predisposition, Labile INR, Elderly, Drug/alcohol usage (HAS-BLED) and congestive heart failure, hypertension, age ≥75 years, diabetes mellitus, stroke or transient ischemic attack, vascular disease, age 65 to 74 years, sex category (CHA2DS2-VASc) components.

Method			HAS-BLED											CHA_2_DS_2_-VASc								
			Structured surveillance		Structured and NLP^a^ surveillance		Difference		*T* test		*P* value		Structured surveillance			Structured and NLP surveillance		Difference		Test statistic		*P* value
**Sensitivity**																						
	McNemar method	0.382		0.806		0.424		72		<.001		—^b^			—		—		—		—	
	Exact binomial method	—		—		—		—				0.942			0.983		0.0413		—		.002	
**Specificity**																						
	McNemar method	0.947		0.777		–0.17		16		<.001												
	Exact binomial method	—		—		—		—		—		0.955			0.909		–0.0455				>.99^c^	
**PPV^d^**																						
	Generalized score method	0.929		0.867		.061		4.487		.03		0.996			0.992		0.004		0.915		.34	
**NPV^e^**																						
	Generalized score method	0.459		0.689		0.23		47.757		<.001		0.6			0.833		0.233		11.662		<.001	

^a^NLP: natural language processing.

^b^There is a small number of discordant cells, such that for the gold standard’s CHA_2_DS_2_-VASc <2, there is 1 case that was identified as CHA_2_DS_2_-VASc ≥2 in the structured and NLP method but not in the structured method. The exact binomial *P* value is calculated as 
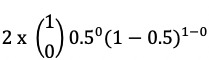

^c^There is a small number of discordant cells, such that for the gold standard’s CHA_2_DS_2_-VASc <2, there is 1 case that was identified as CHA_2_DS_2_-VASc >2 in the Structured and NLP method but not in the structured method. The exact binomial *P* value is calculated as 
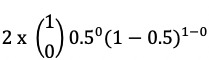

^d^PPV: positive predictive value.

^e^NPV: negative predictive value.

Multimedia Appendix 1, Figure S4 presents the conditional probability tree for the automated structured or structured-plus-NLP method, based on clinical guidelines.

**Figure 1:**
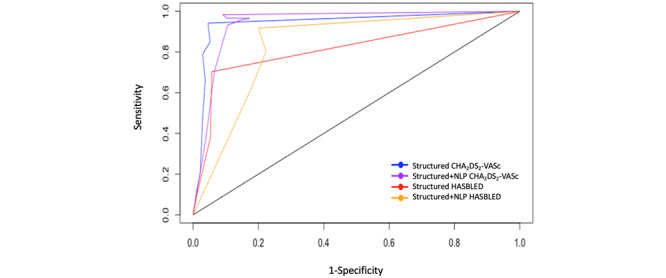
Four receiver operator characteristic curves for cumulative congestive heart failure, hypertension, age ≥ 75 years, diabetes mellitus, stroke or transient ischemic attack, vascular disease, age 65 to 74 years, sex category (CHA2DS2-VASc), and Hypertension, Abnormal liver/renal function, Stroke history, Bleeding history or predisposition, Labile INR, Elderly, Drug/alcohol usage (HAS-BLED) risk scores. NLP: natural language processing.

In Figure 1, the area under the ROC for the CHA_2_DS_2_-VASc scores for the structured-plus-unstructured data compared with the gold standard score was 0.914 (95% CI 0.896-0.933) with a Somer D 0.829 (SD 0.0185), and for the structured data alone compared with the gold standard score, was 0.863 (CI 0.838-0.887), with a Somer D 0.726 (SD 0.0249). For CHA_2_DS_2_-VASc scores, structured-plus-unstructured data were more concordant than structured data alone when compared with the gold standard score (Z=19.77; *P*<.001). For the ROC curves of the HAS-BLED scores with the gold standard score as the outcome, the structured-plus-unstructured data was 0.816 (CI 0.783-0.849), with a Somer D 0.633 (SD 0.034), and the structured data alone was 0.797 (CI 0.761-0.833) with a Somer D 0.595 (SD 0.037). For HAS-BLED scores, structured-plus-unstructured data were not more concordant than structured data alone (Z=1.433; *P*=.149).

Figure 1 represents four areas under ROC curves, two for structured versus structured and NLP CHA_2_DS_2_-VASc score and two for structured versus structured and NLP HAS-BLED score. As these scores are ordinal (eg, ranging from 0-9) and not binary, as with typical ROC, we use the C-Index and Somer D based on ordinal logistic regression to model the probabilities, resulting in multiple y values for the same x.

We compared the findings of the gold standard with the NLP structured-plus-unstructured data (Multimedia Appendix 1, Table S1). Clinician reviewers found 31 untreated patients who should have been treated and 1 treated patient who, the clinicians felt, should not have been treated. This was the same total as that of the gold standard. After clinician review, there was a 32.1% improvement in PPV using the structured-plus-unstructured method when compared with the structured method alone.

### Extrapolating Findings to the US Population for Prevalence and Cost

Extrapolation to the US population of the Truman and Optum data results can be found in Table 3.

To determine the national cost savings from the NLP-assisted bio-surveillance of the structured-plus-unstructured data, we used Truven data and contrasted the mean monthly costs per patient after a stroke (US $11,538) with the monthly costs before a stroke (US $2,763.33), which yielded a mean savings of US $8,776.02. This was adjusted to US $2019 as the data were from 2010 to 2015. This revealed savings of US $8,556.66 per month or yearly savings of US $102,680.

The structured data method identified 1.5% (967,801/63,296,120) of the population as having NVAF. Of those cases, 84.3% (816,240/967,801) had a CHA_2_DS_2_-VASc score of ≥2. These data indicate that 60.7% (495,749/816,240) of these patients were not treated despite the current clinical guidelines. Untreated NVAF patients had a 4.4% (22,021/495,749) annual ischemic stroke risk, and the stroke patients had a 6.0% (1320/22,021) risk of death.

**Table 3:** Optum and Truven stroke data for 1 year after atrial fibrillation (AF) diagnosis.

Population for rates	Truven, n (%)	Optum, n (%)	Total, n (%)	Event rates (%)
All patients	32,046,193 (50.63)	31,249,927 (49.37)	63,296,120 (100)	—^a^
Patients aged ≥18 years in 2016 with any diagnosis of AF during October 2015-September 2016	422,092 (32.79)	865,072 (67.21)	1,287,164 (100)	—
Patients aged ≥18 years in 2016 with any diagnosis of AF during October 2015-September 2016 and without a VHD^b^ diagnosis during 1-year preindex	355,811 (36.76)	611,990 (63.24)	967,801 (100)	1.5
Patients aged ≥18 years in 2016 with any diagnosis of AF during October 2015-September 2016 and without VHD diagnosis during 1-year preindex and with CHA_2_DS_2_‑VASc^c^ ≥2 and no contraindications to OAC^d^	276,465 (33.87)	539,775 (66.13)	816,240 (100)	84.3
Patients aged ≥18 years in 2016 with any diagnosis of AF during October 2015-September 2016 and without VHD diagnosis during 1-year preindex and with CHA_2_DS_2_‑VASc ≥2 and no contraindications to OAC and were untreated	179,441 (36.20)	316,308 (63.80)	495,749 (100)	60.7
Stroke rate	11,530 (52.36)	10491 (47.64)	22,021 (100)	4.4
Death rate	727 (55.1)	593 (44.9)	1,320 (100)	5.99

^a^The values are not events.

^b^VHD: valvular hear disease.

^c^CHA_2_DS_2_‑VASc: congestive heart failure, hypertension, age ≥ 75 years, diabetes mellitus, stroke or transient ischemic attack, vascular disease, age 65 to 74 years, sex category.

^d^OAC: oral anticoagulation.

### Estimates of Morbidity, Mortality, and Cost

After extrapolating our results combining the Optum and Truven data with our method of bio-surveillance, we estimated outcomes of implementing the NLP-assisted analyses of structured-plus-unstructured data nationally; that is, if implemented nationally (among a population of 316,005,000), this system could potentially prevent 176,537 strokes and 10,575 deaths in the first year of implementation, with stroke-associated savings >US $18.126 billion (Table 4).

**Table 4:** Untreated strokes and their costs for first year after the event.

Extrapolated results	Structured surveillance	Structured and NLP^a^ surveillance	Difference between the two methods
NVAF^b^ population	4,955,284	6,545,930	1590,646
NVAF population with no contraindications and CHA_2_DS_2_VASc^c^ ≥2	4,543,995	6,002,707	1,458,712
NVAF population needing treatment	3,009,840	3,976,057	966,217
Strokes prevented	133,637	176,537	42,900
Deaths prevented	8,005	10,575	2,570
Cost savings^d^ (US $)	13,721,820,000	18,126,800,000	4,404,981,210

^a^NLP: natural language processing.

^b^NVAF: nonvalvular atrial fibrillation.

^c^CHA_2_DS_2_-VASc: congestive heart failure, hypertension, age ≥ 75 years, diabetes mellitus, stroke or transient ischemic attack, vascular disease, age 65 to 74 years, sex category.

^d^Cost basis is US $102,680 per untreated ischemic stroke patient's excess cost for the first year after event; cost is 1.9% inflation adjusted.

## Discussion

### Principal Findings

Compared with structured EHR data alone, we found that NLP-assisted structured-plus-unstructured EHR data identified previously unknown and untreated patients with NVAF and their stroke and bleed risks with greater accuracy. Adding the unstructured data significantly improved the sensitivity and negative predictive value across all measures, whereas the results for NVAF specificity and PPV were strong but mixed. Future applications of this artificial intelligence (AI) bio-surveillance method may involve identifying other underdiagnosed populations.

We estimated NVAF rates in large national database populations, the percentage of people who should be treated with OAC and are not currently treated, and yearly risks of stroke expressed as a percentage of these untreated patients [24,25]. We also estimated the average incremental 1-year cost for a stroke event and identified stroke-related average death rates in the first year after event.

Verhoef et al [26,27] showed that bleeding rates with warfarin were, on average, 0.34% risk per year. Given additional treatment for 3,976,057 new patients, we would expect 13,824 new patient bleeds. McWilliam [28] showed that the average cost of a major bleed was US $19,000 in 2008 (inflation adjusted to US $23,777.67). For the population, this equals US $328,702,452. Gilligan et al [29] showed that the average total cost for warfarin therapy was US $76.19 per member per month, which translates to a total national cost of US $3,750,758,790 per year. Potential net financial treatment benefits from using the NLP-assisted structured-plus-unstructured method equates to US $14.4 billion (US $18.13 billion to US $3.75 billion).

On the basis of the accuracy of the AI-derived bio-surveillance method, we show potential societal benefits of implementing this technology. Nationally, this method could identify approximately 4 million patients requiring treatment, potentially preventing >176,000 strokes in the first year, and >10,500 deaths, translating to national savings of >US $14 billion. Including the estimated costs of excess bleeding from the treatment and from our estimate, the national implementation costs would be no greater than US $300,000,000. This type of AI-driven clinical decision support bio-surveillance has the potential to significantly improve patient care and clinicians’ treatment decisions.

NVAF is but one important condition among many. Future applications of this AI bio-surveillance method may identify other underdiagnosed populations. Once deployed, the infrastructure could be used for other disorders and could be implemented at a low incremental cost.

### Limitations

This analysis and data extrapolation were based on previous 2014 American Heart Association, American College of Cardiology, and Heart Rhythm Society recommendations for OAC therapy in patients with NVAF and a CHA_2_DS_2_-VASc score of ≥2. The 2019 focused updates on AF now recommend that men with a CHA_2_DS_2_-VASc score of ≥2 and women with a CHA_2_DS_2_-VASc score of ≥3 should be treated with an OAC. As such, the numbers in this analysis may include women who, under the updated guidance, may not be recommended for treatment with an OAC. In addition, not all patients for whom therapy is indicated may agree to accept anticoagulation therapy.

The Optum and Truven databases, although found to be effectively nonoverlapping, are, on average, considered to be for younger and healthier private payer populations; therefore, we may underestimate both protective effects and cost savings [30]. If this method were extended to other diseases, models must be built and distributed uniformly across the country and perhaps internationally.

The AI model processes the free text of the notes and reports, and as it can accept and process data from Cerner, Epic, and other EHRs, there should be no difference in outcome; however, this model has not been specifically tested with data from other EHRs.

ICD-9 codes were used in this study because of the desire to have a consistently coded data set. ICD-10 codes were not included. Future research should investigate this method using later ICD codes.

This informatics method promises many benefits. Of course, additional research is needed to determine its applicability to other diseases.

### Conclusions

Although a common disorder (N=6 million Americans), NVAF is often underprophylaxed for thromboembolic events that may lead to strokes. Critical evidence may be found in patients’ EHRs to aid in anticoagulation decision-making. Stroke rates of untreated patients with a CHA_2_DS_2_‑VASc of ≥2 in our study were 4.44%, and of these, approximately 6% will die within 1 year. Treatment dramatically reduces one’s odds of a stroke to <0.5% on average.

Our structured-plus-unstructured (NLP) method identified 36.3% additional true NVAF cases (*P*<.001) compared with the structured data alone. Extrapolating to the US population using the 63 million people in the Optum and Truven populations allowed us to predict that in just the first-year implementation of this system, it could prevent 176,537 strokes and 10,575 deaths and save the nation >US $13.5 billion dollars.

Moreover, this bio-surveillance method and preparedness, in general, may be useful for the discovery and treatment of many other disorders, and require further research with different diseases. Automated tools in partnership with clinicians have the potential to significantly improve adherence to established clinical guidelines and to precision medicine.

## Appendix

Multimedia Appendix 1 Study recruitment diagram and additional analyses.

Multimedia Appendix 2 Financial and nonfinancial support.

## Abbreviations

AF: atrial fibrillation
AI: artificial intelligence
CHA2DS2‑VASc: congestive heart failure, hypertension, age ≥ 75 years, diabetes mellitus, stroke or transient ischemic attack, vascular disease, age 65 to 74 years, sex category
EHR: electronic health record
HAS-BLED: Hypertension, Abnormal liver/renal function, Stroke history, Bleeding history or predisposition, Labile INR, Elderly, Drug/alcohol usage
HD-NLP: high definition natural language processing
ICD: International Classification of Disease
NIH: National Institutes of Health
NLP: natural language processing
NVAF: nonvalvular atrial fibrillation
OAC: oral coagulation
OR: odds ratio
PPV: positive predictive value
ROC: receiver operator characteristic curve
SNOMED CT: Systematized Nomenclature of Medicine–Clinical Terms
VAF: valvular atrial fibrillation

## References

[1] Camm AJ, Lip GY, De Caterina R, Savelieva I, Atar D, Hohnloser SH, Hindricks G, Kirchhof P, ESC Committee for Practice Guidelines (CPG) (2012). 2012 focused update of the esc guidelines for the management of atrial fibrillation: an update of the 2010 esc guidelines for the management of atrial fibrillation. Developed with the special contribution of the european heart rhythm association. Eur Heart J.

[2] January CT, Wann LS, Alpert JS, Calkins H, Cigarroa JE, Cleveland JC, Conti JB, Ellinor PT, Ezekowitz MD, Field ME, Murray KT, Sacco RL, Stevenson WG, Tchou PJ, Tracy CM, Yancy CW, American College of Cardiology/American Heart Association Task Force on Practice Guidelines (2014). 2014 AHA/ACC/HRS guideline for the management of patients with atrial fibrillation: a report of the american college of cardiology/american heart association task force on practice guidelines and the heart rhythm society. J Am Coll Cardiol.

[3] Roger VL, Go AS, Lloyd-Jones DM, Benjamin EJ, Berry JD, Borden WB, Bravata DM, Dai S, Ford ES, Fox CS, Fullerton HJ, Gillespie C, Hailpern SM, Heit JA, Howard VJ, Kissela BM, Kittner SJ, Lackland DT, Lichtman JH, Lisabeth LD, Makuc DM, Marcus GM, Marelli A, Matchar DB, Moy CS, Mozaffarian D, Mussolino ME, Nichol G, Paynter NP, Soliman EZ, Sorlie PD, Sotoodehnia N, Turan TN, Virani SS, Wong ND, Woo D, Turner MB, American Heart Association Statistics Committee and Stroke Statistics Subcommittee (2012). Heart disease and stroke statistics--2012 update: a report from the American Heart Association. Circulation.

[4] Wolf PA, Abbott RD, Kannel WB (1991). Atrial fibrillation as an independent risk factor for stroke: the framingham study. Stroke.

[5] Gage BF, Waterman AD, Shannon W, Boechler M, Rich MW, Radford MJ (2001). Validation of clinical classification schemes for predicting stroke: results from the national registry of atrial fibrillation. J Am Med Assoc.

[6] Lip GY, Nieuwlaat R, Pisters R, Lane DA, Crijns HJ (2010). Refining clinical risk stratification for predicting stroke and thromboembolism in atrial fibrillation using a novel risk factor-based approach: The Euro Heart Survey on atrial fibrillation. Chest.

[7] January CT, Wann LS, Calkins H, Chen LY, Cigarroa JE, Cleveland JC, Ellinor PT, Ezekowitz MD, Field ME, Furie KL, Heidenreich PA, Murray KT, Shea JB, Tracy CM, Yancy CW (2019). 2019 AHA/ACC/HRS focused update of the 2014 AHA/ACC/HRS guideline for the management of patients with atrial fibrillation: a report of the american college of cardiology/american heart association task force on clinical practice guidelines and the heart rhythm society in collaboration with the society of thoracic surgeons. Circulation.

[8] Pisters R, Lane DA, Nieuwlaat R, de Vos CB, Crijns HJ, Lip GY (2010). A novel user-friendly score (has-bled) to assess 1-year risk of major bleeding in patients with atrial fibrillation: the euro heart survey. Chest.

[9] Lip GY, Frison L, Halperin JL, Lane DA (2011). Comparative validation of a novel risk score for predicting bleeding risk in anticoagulated patients with atrial fibrillation: the has-bled (hypertension, abnormal renal/liver function, stroke, bleeding history or predisposition, labile inr, elderly, drugs/alcohol concomitantly) score. J Am Coll Cardiol.

[10] Tiryaki F, Nutescu EA, Hennenfent JA, Karageanes AM, Koesterer LJ, Lambert BL, Schumock GT (2011). Anticoagulation therapy for hospitalized patients: patterns of use, compliance with national guidelines, and performance on quality measures. Am J Health Syst Pharm.

[11] Navar-Boggan AM, Rymer JA, Piccini JP, Shatila W, Ring L, Stafford JA, Al-Khatib SM, Peterson ED (2015). Accuracy and validation of an automated electronic algorithm to identify patients with atrial fibrillation at risk for stroke. Am Heart J.

[12] Demner-Fushman D, Chapman WW, McDonald CJ (2009). What can natural language processing do for clinical decision support?. J Biomed Inform.

[13] Aakre C, Dziadzko M, Keegan MT, Herasevich V (2017). Automating clinical score calculation within the electronic health record. A feasibility assessment. Appl Clin Inform.

[14] Elkin PL, Froehling DA, Wahner-Roedler DL, Brown SH, Bailey KR (2012). Comparison of natural language processing biosurveillance methods for identifying influenza from encounter notes. Ann Intern Med.

[15] Yeung S, Downing NL, Fei-Fei L, Milstein A (2018). Bedside computer vision - moving artificial intelligence from driver assistance to patient safety. N Engl J Med.

[16] Murff HJ, FitzHenry F, Matheny ME, Gentry N, Kotter KL, Crimin K, Dittus RS, Rosen AK, Elkin PL, Brown SH, Speroff T (2011). Automated identification of postoperative complications within an electronic medical record using natural language processing. J Am Med Assoc.

[17] Schlegel DR, Crowner C, Lehoullier F, Elkin PL (2017). HTP-NLP: A new NLP system for high throughput phenotyping. Stud Health Technol Inform.

[18] Birman-Deych E, Waterman AD, Yan Y, Nilasena DS, Radford MJ, Gage BF (2005). Accuracy of ICD-9-CM codes for identifying cardiovascular and stroke risk factors. Med Care.

[19] Leisenring W, Alonzo T, Pepe MS (2000). Comparisons of predictive values of binary medical diagnostic tests for paired designs. Biometrics.

[20] (2019). Optum.

[21] (2019). Truven Health Analytics. IBM.

[22] Wade G (2011). Implementing snomed ct for quality reporting: avoiding pitfalls. Appl Clin Inform.

[23] Bandrowski A, Brinkman R, Brochhausen M, Brush MH, Bug B, Chibucos MC, Clancy K, Courtot M, Derom D, Dumontier M, Fan L, Fostel J, Fragoso G, Gibson F, Gonzalez-Beltran A, Haendel MA, He Y, Heiskanen M, Hernandez-Boussard T, Jensen M, Lin Y, Lister AL, Lord P, Malone J, Manduchi E, McGee M, Morrison N, Overton JA, Parkinson H, Peters B, Rocca-Serra P, Ruttenberg A, Sansone S, Scheuermann RH, Schober D, Smith B, Soldatova LN, Stoeckert CJ, Taylor CF, Torniai C, Turner JA, Vita R, Whetzel PL, Zheng J (2016). The ontology for biomedical investigations. PLoS One.

[24] Bray BD, Paley L, Hoffman A, James M, Gompertz P, Wolfe CD, Hemingway H, Rudd AG (2018). Socioeconomic disparities in first stroke incidence, quality of care, and survival: a nationwide registry-based cohort study of 44 million adults in england. Lancet Public Health.

[25] Freedman B, Potpara TS, Lip GY (2016). Stroke prevention in atrial fibrillation. Lancet.

[26] Verhoef TI, Redekop WK, Darba J, Geitona M, Hughes DA, Siebert U, de Boer A, Maitland-van der Zee A, Barallon R, Briz M, Daly A, Haschke-Becher E, Kamali F, Kirchheiner J, Manolopoulos VG, Pirmohamed M, Rosendaal FR, van Schie RM, Wadelius M, EU-PACT Group (2010). A systematic review of cost-effectiveness analyses of pharmacogenetic-guided dosing in treatment with coumarin derivatives. Pharmacogenomics.

[27] Yao X, Abraham NS, Sangaralingham LR, Bellolio MF, McBane RD, Shah ND, Noseworthy PA (2016). Effectiveness and safety of dabigatran, rivaroxaban, and apixaban versus warfarin in nonvalvular atrial fibrillation. J Am Heart Assoc.

[28] Desai JR, Hyde CL, Kabadi S, St Louis M, Bonato V, Loomis AK, Galaznik A, Berger ML (2017). Utilization of positive and negative controls to examine comorbid associations in observational database studies. Med Care.

[29] McWilliam A, Lutter R, Nardinelli C (2008). Healthcare impact of personalized medicine using genetic testing: an exploratory analysis for warfarin. Per Med.

[30] Gilligan AM, Gandhi P, Song X, Wang C, Henriques C, Sander S, Smith DM (2017). All-cause, stroke-, and bleed-specific healthcare costs: comparison among patients with Non-Valvular Atrial Fibrillation (NVAF)newly treated with dabigatran or warfarin. Am J Cardiovasc Drugs.

