# Supplementary material for: Using Artificial Intelligence With Natural Language Processing to Combine Electronic Health Record’s Structured and Free Text Data to Identify Nonvalvular Atrial Fibrillation to Decrease Strokes and Death: Evaluation and Case-Control Study
Source: J Med Internet Res. 2021 Nov 9;23(11):e28946. doi: 10.2196/28946 (PMC8663460; doi:10.2196/28946)
Supplement: Multimedia Appendix 1 [file jmir_v23i11e28946_app1.docx]

**Supplemental Figures and Tables**

Figure A: Patient Inclusion/Exclusion Criteria

Figure B: Randomization Scheme

Figure C: NLP Workflow Process and accompanying text

Figure D: Conditional probability tree for Automated Method (Structured or Structured plus NLP) based on Clinical Guidelines.

Table A: Clinician Judgement of Patient’s Benefit versus Risk of OAC Use by Risk Score for Each Method

Table B: Variable definitions used in the project.

Table C: The SNOMED-CT code retained for inclusion criteria

Table D: Exclusion Codes

Table E: ICD9 codes utilized

Table F: RxNorm Codes Utilized

Supplemental Figure A: Patient Inclusion/Exclusion Criteria (next page)

Notes for Supplemental Figure A, Above. Patient Inclusion/Exclusion Criteria.

The figure presents the decision tree and sample numbers.

Participants 18 through 90 years old were included. For population level statistics, we analyzed data from 63,296,120 participants in the Optum and Truven databases to determine the frequency of NVAF, rates of CHA_2_DS_2_‑VASc >= 2 and no contraindications to Oral Anticoagulants (OAC), rates of stroke and death in the untreated population, and first year’s cost after stroke.^1,2^

We employ a high throughput phenotyping-natural language processing system (HTP-NLP) to codify the notes. We then compare the accuracy of structured data alone to structured-plus-unstructured EHR data derived using the HTP-NLP system. Participants were retrospectively collected from Allscripts EHR data. The supervised machine learning algorithm training set used 36,268 patients with 1972 cases of Afib and 1795 cases of NVAF^.3,4,5^ The testing set came from Allscripts outpatient EHR data from UBMD faculty practices, (N = ~ 500,000 patients). The research was approved by the WNY IRB.

Patient data were abstracted from 2010 through 9/21/2015--prior to the switch to ICD-10-- allowing consistent use of ICD-9 terminology; yielding 212,343 patients. Of those, 96,681 had notes and were seen for ≥1outpatient visit. Outcomes from these data included rates of AF, NVAF, and VAF diagnosis, components of the CHA_2_DS_2_-VASc and HAS-BLED scores, relevant contraindications, OAC treatment, and demographic variables. We removed patients on oral antithrombotic therapy for an indication other than NVAF, had a mechanical prosthetic valve, had a hemodynamically significant mitral stenosis or aortic stenosis, were pregnant, had a transient AF due to reversible conditions, or if they had an active infective endocarditis.

After removing false positive and false negative cases from the dataset, we then added additional synonymy to the terminology and selected more appropriate set of codes for each rule. The two models were compared in their ability to identify true cases of NVAF and to determine stroke and bleeding risks (CHA_2_DS_2_-VASc and HAS-BLED Scores).

References: Supplemental Figure A

1. <https://www.optum.com/solutions/data-analytics/data.html> (Accessed 2/2/2019)
2. <https://truvenhealth.com/Portals/0/assets/ACRS_11223_0912_MarketScanResearch_SS_Web.pdf> (Accessed 2/2/2019)
3. Elkin PL, Froehling D, Wahner-Roedler D, Brown SH, Bailey K. “Comparison of NLP Biosurveillance Methods for Identifying Influenza from Encounter Notes”; Ann Intern Med. 2012 Jan 3;156(1 Pt 1):11-8.
4. Murff HJ, FitzHenry F, Matheny ME, Gentry N, Kotter KL, Crimin K, Dittus RS, Rosen AK, Elkin PL, Brown SH, Speroff T. [Automated identification of postoperative complications within an electronic medical record using natural language processing.](http://www.ncbi.nlm.nih.gov/pubmed/21862746) JAMA. 2011 Aug 24;306(8):848-55.
5. Schlegel DR, Crowner C, Lehoullier F, Elkin PL. HTP-NLP: A New NLP System for High Throughput Phenotyping. Stud Health Technol Inform. 2017;235:276-280.

Supplemental Figure B. Randomization Scheme


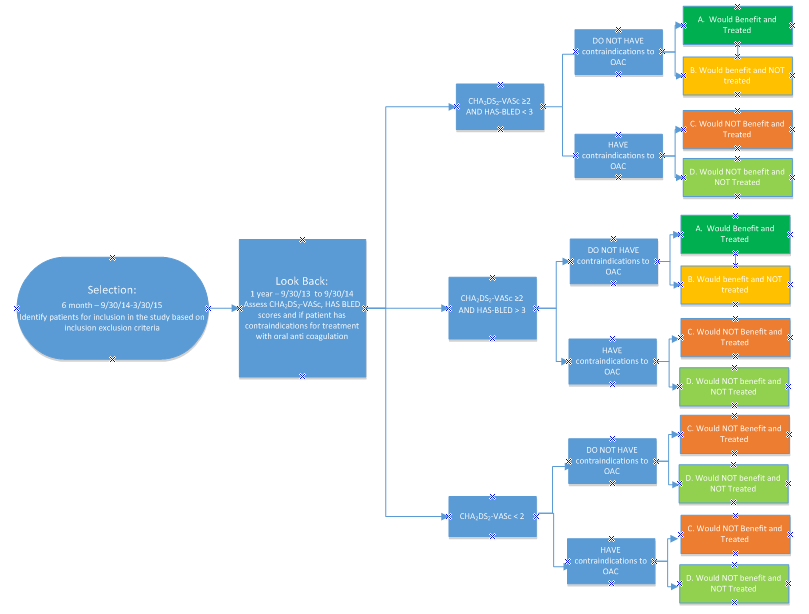


For validation of the process, we used a gold standard created by human review from a random sample of 300 patients. Calculations determined 300 patients were needed for 90% power to predict a five percent change in accuracy given a two‑sided alpha of 0.05; assuming a standard accuracy of 73% based on ICD-9 codes.^21^ We obtained a random sample to compare structured to structured-plus-unstructured EHR data. We used codes Atrial Fibrillation (ICD-9 427.31) or Atrial Flutter (ICD-9 427.32) and SNOMED CT codes (and SNOMED CT codes (49436004, 5370000 respectively with all subtypes in the hierarchy).

The human review dataset was independently examined by two clinicians, each performing 150 reviews on de-identified patient encounters from the EHR. If there were disagreements, a third clinician adjudicated.

The structured data only method used ICD-9 codes from problem lists, medications, and demographics. The structured-plus-unstructured method added the clinical notes parsed with HTP-NLP, the vital signs, laboratory findings, and text from the problem list. Free text elements were coded using SNOMED-CT, a general description logic based nomenclature of clinical medicine. No cases were identified by the structured method which were missed by the unstructured method.

After removing false positive and false negative cases from the dataset, we then added additional synonymy to the terminology and selected more appropriate set of codes for each rule. The two models were compared in their ability to identify true cases of NVAF and to determine stroke and bleeding risks (CHA_2_DS_2_-VASc and HAS-BLED Scores).

We used a supervised machine learning algorithm to generate the SNOMED CT models which were then tested on a separate population of patients (training set / test set methodology). A population of 36,268 patients was selected as our training set to identify the codes to use in the SNOMED CT model.

There were 1972 cases of Afib and 1795 cases of NVAF in the training set, the resultant SNOMED CT model was used in the structured plus unstructured method in our test set of 96,681 patients. Once all the false positive and false negative cases were reviewed, interventions were taken like adding additional synonymy to the terminology or choosing a more appropriate set of codes for each rule. The two models were compared in terms of their ability to identify true cases of NVAF and to determine the CHA_2_DS_2_-VASc, which has been shown to predict stroke risk, and HAS-BLED Scores, which has been shown to predict bleeding risk on oral anticoagulants.


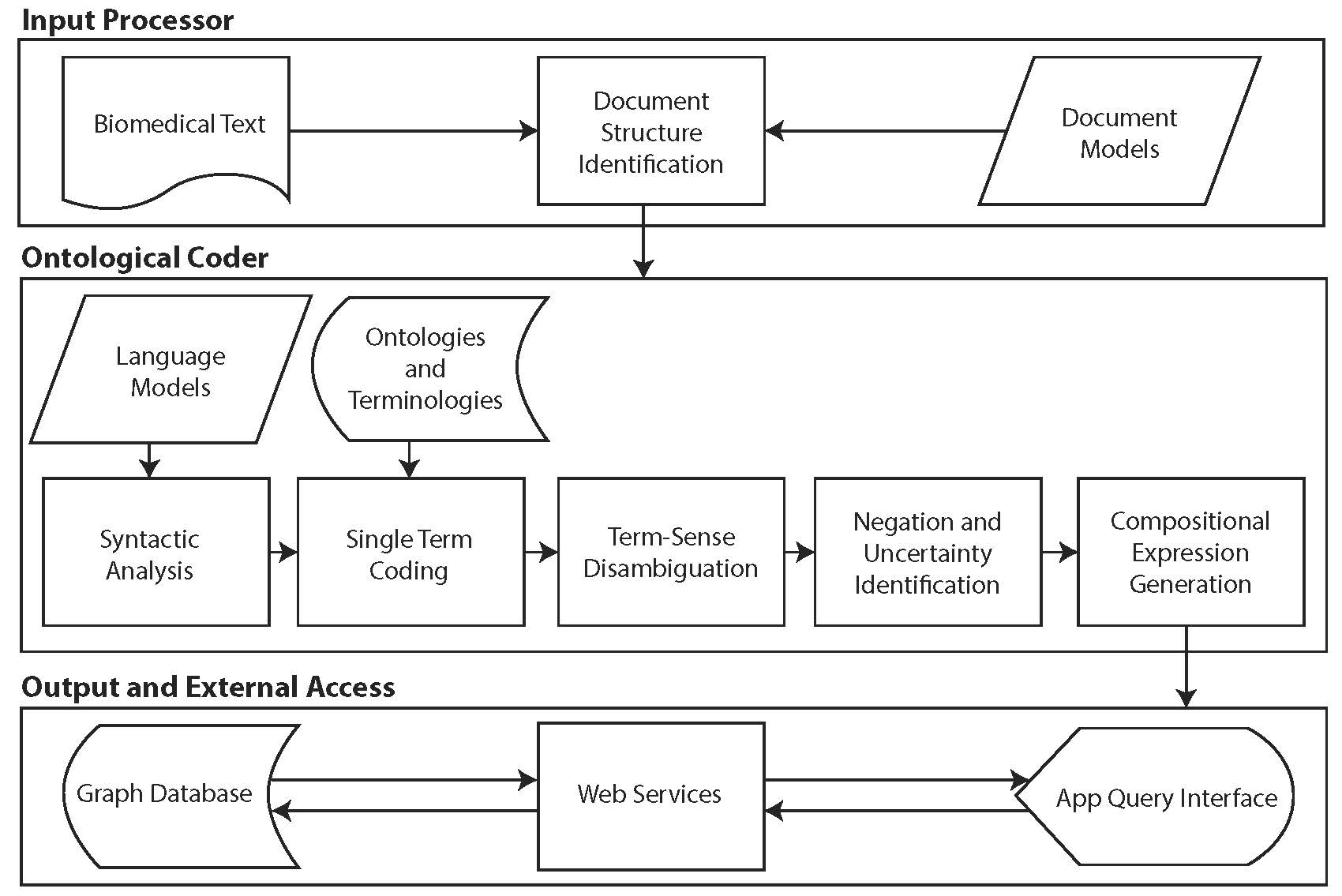
Supplemental Figure C NLP Workflow Process and accompanying text

We describe the transformations which were applied to the clinical data in selecting the cohort:

1. **Raw Data Extract** The following tables were exported from the live Allscripts Analytics SQL server which are of interest to this study: Allergies, Encounters, Findings, Immunizations, Medications, Clinical Notes, Orders, Patients, Problems, Results. During this process the data were anonymized by generating research IDs in place of patient IDs. Also, identifiable patient data (eg, name and address) were excluded.
2. **Local SQL Server** The raw data were imported into an instance of SQL server on our development machine.
3. **BerkeleyDB** The text fields from Allscripts were extracted and placed in a Berkeley Database keyed on research ID, encounter ID, and section name. This database is used to populate the Notes section of the clinical note viewer for clinician assessment and creation of the gold standard, as well as for the NLP process.
4. **BerkeleyDBs for NLP and Cohort Selection** The NLP process ingests data from BerkeleyDBs. The Problem and Orders tables also have their textual content stored in BerkeleyDBs for NLP, and contain other data (such as ICD-9 codes) for cohort selection.
5. **BerkeleyDBs for NLP Output** The output of the NLP process is a BerkeleyDB containing, all data items pertinent for the study.

When ontology terms matched to free text notes, it is the goal to select the term which has the meaning intended by the author of the original text. Term Sense Disambiguation (TSD) is the process by which a single term is selected from several which cover the same span of text in the input document. To do TSD we use the ontology in which the ambiguous terms are present. Excluding the ISA (hierarchical) relation (i.e. NVAF ISA Afib ISA Dysrhythmia ISA Cardiovascular disorder), we attempt to find a common ancestor in the ontology of each codified word in the surrounding text, and each candidate ambiguous term in turn. The term which is most related to the surrounding text is selected.

Negated and uncertain phrases are identified using a modified version of the NegEx algorithm^1^ which is capable of identifying not only negated words and phrases but negated subwords (eg, “steroidal” in “nonsteroidal”).

Compositional Expressions are identified using a database of noun phrase surface feature to graph transformations, where the nodes in the graph are terms, and the edges are relations.

*Natural Language Processing*

We make use of a high throughput phenotyping system to rapidly assign ontology terms to text in patient records. We used the Basic Formal Ontology as an upper level ontology to index the data from individual trials. We also used the Ontology of Biomedical Investigation and SNOMED-CT as our main ontologies.

A level of syntactic processing is required to match text with ontological terms. The linguistic representation is specified in language models. Of primary concern here is an English language model to identify sentences, phrases, words, and parts of speech. Terms from the input ontologies are then assigned to spans of text. Using string matching techniques allows for inexact matches influenced by the underlying language model. The structure of free‑text medical records is captured and stored.

*Statistical Analysis*

Statistical analysis used R 3.3.2. For the random gold standard sample of 300 cases, inter-rater agreement was assessed using the 2-way random effects model for intraclass correlation coefficient (ICC) with two-sided 10,000 samples bootstrapped 95% confidence interval, treating the risk scores as continuous. Cohen’s kappa with two-sided 10,000 samples bootstrapped 95% confidence interval assessed the inter-rater reliability of each individual component of the scores, NVAF, and AF.

References: Supplemental Figure C

## 1. Chapman WW, Bridewell W, Hanbury P, [Cooper](https://www.sciencedirect.com/science/article/pii/S1532046401910299#!) GF, Buchanan BG. Simple Algorithm for Identifying Negated Findings and Diseases in Discharge Summaries [Journal of Biomedical Informatics](https://www.sciencedirect.com/science/journal/15320464) [Volume 34, Issue 5](https://www.sciencedirect.com/science/journal/15320464/34/5), October 2001, Pages 301-310

**Supplemental Figure D Conditional Probability Tree for Automated Method (Structured or Structured plus NLP) based on Clinical Guidelines.**


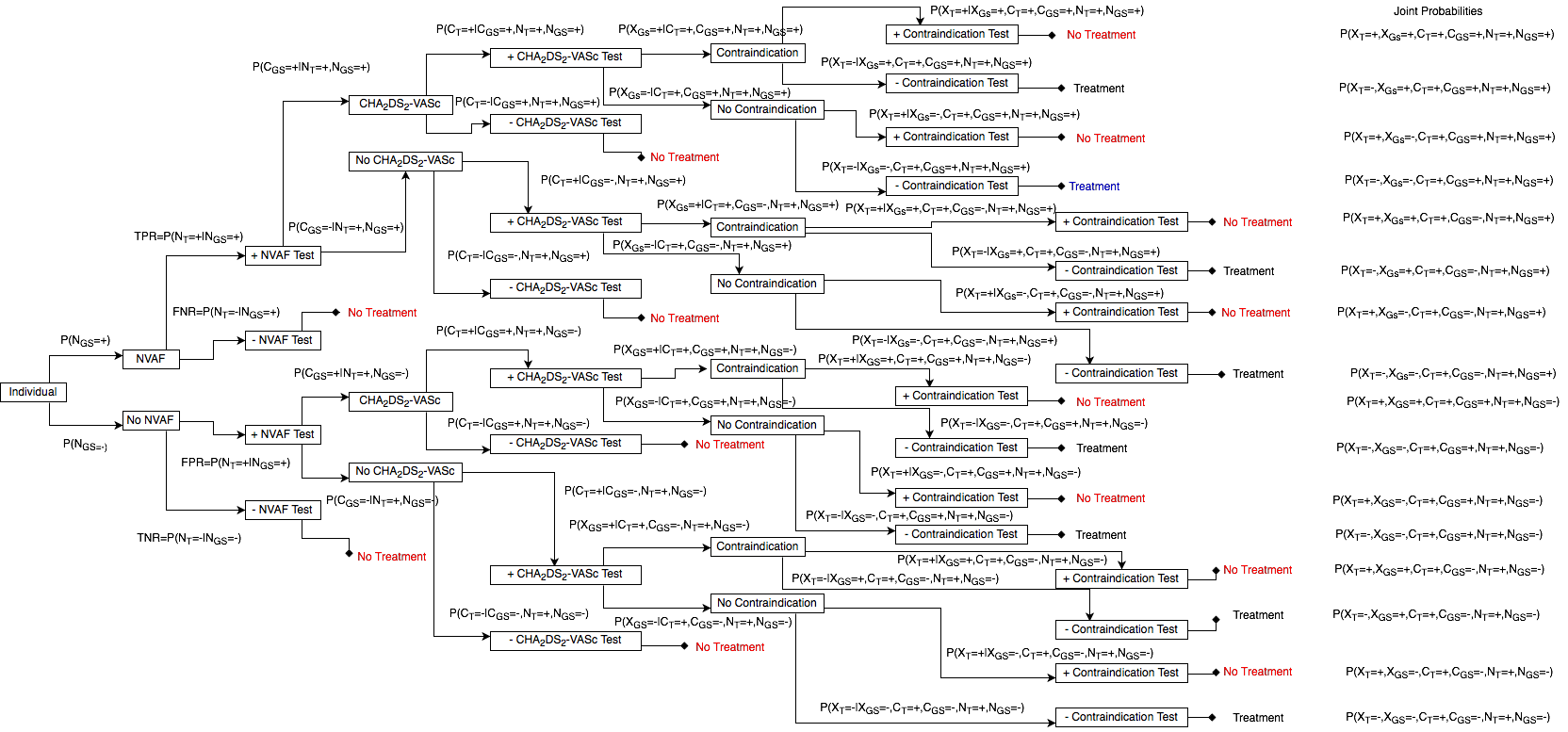


Each end node has an outcome: treatment or no treatment based on the automated method. No Treatment indicates that the automated method was negative for at least one outcome. Treatment indicates that the automated method and gold standard agree that the patients should be treated. Probability outcomes are defined by the stratified random sample of 300 cases. Treatment does not mean that cases should or should not be treated, but that the automated method has classified them as ‘treatment’ or ‘no treatment’.

Comparing the two methods using the decision tree above, we found that the Structured method would accurately identify for treatment 57.7% of the population and the Structured plus Unstructured method would treat 86.3% of the population accurately (p<0.001, McNemar Chi-Square Test).

**Supplemental Table A Clinician Judgement of Patient’s Benefit versus Risk of OAC use by Risk Score for Each Method**

|  |  | Would Benefit and On OAC | Would Benefit and Not on OAC | Would Not Benefit and Are on OAC | Would Not Benefit and Are Not on OAC |
| --- | --- | --- | --- | --- | --- |
| Gold Standard with Contraindication | *CHA_2_DS_2_-VASc >2 AND HAS-BLED <3 and Contraindication* | 3 | 2 | 0 | 1 |
|  | *CHA_2_DS_2_-VASc > 2AND HAS-BLED ≥ 3 and Contraindication* | 6 | 0 | 0 | 1 |
|  | *CHA_2_DS_2_-VASc <2 and Contraindication* | 0 | 0 | 0 | 1 |
| Gold Standard with No Contraindication | *CHA_2_DS_2_-VASc >2 AND HAS-BLED <3 and No Contraindication* | 38 | 15 | 0 | 14 |
|  | *CHA_2_DS_2_-VASc >2 AND HAS-BLED ≥ 3 and No Contraindication* | 129 | 16 | 1 | 16 |
|  | *CHA_2_DS_2_-VASc <2 and No Contraindication* | 10 | 3 | 0 | 8 |
| Structured with Contraindication | *CHA_2_DS_2_-VASc >2 AND HAS-BLED <3 and Contraindication* | 4 | 1 | 0 | 0 |
|  | *CHA_2_DS_2_-VASc >2 AND HAS-BLED ≥ 3 and Contraindication* | 3 | 1 | 0 | 0 |
|  | *CHA_2_DS_2_-VASc <2 and Contraindication* | 0 | 0 | 0 | 0 |
| Structured with No Contraindication | *CHA_2_DS_2_-VASc >2 AND HAS-BLED <3 and No Contraindication* | 109 | 25 | 0 | 21 |
|  | *CHA_2_DS_2_-VASc >2 AND HAS-BLED ≥ 3 and No Contraindication* | 49 | 5 | 0 | 11 |
|  | *CHA_2_DS_2_-VASc <2 and No Contraindication* | 21 | 4 | 1 | 8 |
| Structured+NLP with Contraindication | *CHA_2_DS_2_-VASc >2 AND HAS-BLED <3 and Contraindication* | 2 | 0 | 0 | 1 |
|  | *CHA_2_DS_2_-VASc > AND HAS-BLED ≥ 3 and Contraindication* | 6 | 2 | 0 | 1 |
|  | *CHA_2_DS_2_-VASc <2 and Contraindication* | 0 | 0 | 0 | 0 |
| Structured+NLP with No Contraindication | *CHA_2_DS_2_-VASc >2 AND HAS-BLED <3 and No Contraindication* | 53 | 17 | 1 | 8 |
|  | *CHA_2_DS_2_-VASc >2 AND HAS-BLED ≥ 3 and No Contraindication* | 113 | 13 | 0 | 23 |
|  | *CHA_2_DS_2_-VASc <2 and No Contraindication* | 12 | 4 | 0 | 8 |

We compare findings of the gold standard with the NLP’s structured-plus-unstructured data. Clinician reviewers found 31 patients are untreated and should be treated, and one patient was treated who the clinician’s felt should not have been treated. This was the same total as the gold standard. For this study sample, only 13.6% of the cases who should be treated as per the ACC / AHA guidelines were untreated.

The accuracy of the structured data alone was compared to structured-plus-unstructured for the outcomes NVAF, CHA_2_DS_2_-VASc score and HAS-Bled score in the random sample. Cohen’s kappa two-sided confidence intervals were computed using bootstrap methods with 10,000 trials was also calculated as a measure of reliability or agreement between the gold standard and the structured and structured-plus-unstructured. For sensitivity and specificity, a hypothesis test comparing structured to structured-plus-unstructured was assessed either using the McNemar test for paired observations or the binomial exact test.

## *Extrapolating to the population*: For population level statistics, of the 63,296,120 patients from this on average younger and healthier private payer population, 1.52% of the population was identified using the structured model as having NVAF. 84.34% of those cases had a CHA_2_DS_2_-VASc score of > 2. However, 60.74% of those patients were not treated in spite of current clinical guidelines. Untreated NVAF patients had 4.44% annual risk stroke and those stroke patients had a 5.99% risk of death. Each Stroke on average cost the healthcare system an additional $99,041 in the year following the stroke (including the acute stroke visit). **Supplementary Table B. Variables: The following table outlines the variables that will be collected and used for this study. The sources of the codes for the operational definition are provided. Unstructured data operational definitions will be created using the NLP process outlined in Section 7.5.**

| **Variable** | **Role** | **Data Source(s)** | **Operational Definition** | | |
| --- | --- | --- | --- | --- | --- |
| **Demographics** |  |  | **Structured** | **+Unstructured (NLP)**  **(other ontologies as needed)** | **Gold Standard** |
| Age | Risk Score; Pop Characteristics | Allscripts EHR | Demographics |  | 2 clinician review |
| Gender | Risk Score; Pop Characteristics | Allscripts EHR | Demographics |  | 2 clinician review |
| Race | Pop Characteristics | Allscripts EHR | Demographics |  | 2 clinician review |
| Ethnicity | Pop Characteristics | Allscripts EHR | Demographics |  | 2 clinician review |
| Diagnosis of Atrial Fibrillation | Objective 1 | Allscripts EHR | ICD | SNOMED CT | 2 clinician review |
| **CHA2DS2-VASc:** Presence of: |  |  |  |  |  |
| History of Congestive heart failure/Left ventricular dysfunction? | Objective 2a | Allscripts EHR | ICD | SNOMED CT | 2 clinician review |
| History of Hypertension or high blood pressure? | Objective 2a | Allscripts EHR | ICD | SNOMED CT; Vital Signs | 2 clinician review |
| Age ≥ 75? | Objective 2a | Allscripts EHR |  | Demographics | 2 clinician review |
| History of Diabetes mellitus? | Objective 2a | Allscripts EHR | ICD | SNOMED CT; Lab | 2 clinician review |
| History of Stroke/TIA/Thromboembolism? | Objective 2a | Allscripts EHR | ICD | SNOMED CT | 2 clinician review |
| History of Vascular disease? | Objective 2a | Allscripts EHR | ICD | SNOMED CT | 2 clinician review |
| Age 65-74? | Objective 2a | Allscripts EHR | Demographics |  | 2 clinician review |
| Gender category Female? | Objective 2a | Allscripts EHR | Demographics |  | 2 clinician review |
| **HAS-BLED** |  |  |  |  |  |
| Hypertension history? | Objective 2b | Allscripts EHR | ICD | SNOMED CT; Vital Signs | 2 clinician review |
| Renal Disease? | Objective 2b | Allscripts EHR | ICD | SNOMED CT; Lab | 2 clinician review |
| Liver Disease? | Objective 2b | Allscripts EHR | ICD | SNOMED CT; Lab | 2 clinician review |
| Stroke history? | Objective 2b | Allscripts EHR | ICD | SNOMED CT | 2 clinician review |
| Prior Major Bleeding or Predisposition to Bleeding? | Objective 2b | Allscripts EHR | ICD | SNOMED CT | 2 clinician review |
| Labile INR? | Objective 2b | Allscripts EHR | ICD | Lab, LOINC | 2 clinician review |
| Age ≥ 65 years? | Objective 2b | Allscripts EHR | Demographics |  | 2 clinician review |
| Medication Usage Predisposing to Bleeding? | Objective 2b | Allscripts EHR | CPT and Meds | RXNorm; SNOMED | 2 clinician review |
| **Treatment** |  |  |  |  |  |
| Treated with OAC | Objectives 3 and 4 | Allscripts EHR | CPT and Meds | RXNorm; SNOMED | 2 clinician review |
| **Contraindications** |  |  |  |  |  |
| Major surgical procedure within 30 days | Objectives 3 and 4 | Allscripts EHR | CPT; ICD | SNOMED CT | 2 clinician review |
| Clinically significant GI bleed within last 6 mo | Objectives 3 and 4 | Allscripts EHR | ICD; CPT | SNOMED CT; Lab | 2 clinician review |
| Hemorrhagic Disorder or Bleeding Diathesis | Objectives 3 and 4 | Allscripts EHR | ICD | SNOMED CT; Lab | 2 clinician review |
| Known intracranial neoplasm, AVM or aneurysms | Objectives 3 and 4 | Allscripts EHR | ICD | SNOMED CT | 2 clinician review |
| History of intracranial, intraocular, spinal or non-traumatic intraarticular bleeding | Objectives 3 and 4 | Allscripts EHR | ICD | SNOMED CT; Lab | 2 clinician review |
| Current intraarticular bleeding | Objectives 3 and 4 | Allscripts EHR | ICD | SNOMED CT | 2 clinician review |
| Any active bleed | Objectives 3 and 4 | Allscripts EHR | ICD | SNOMED CT; Lab | 2 clinician review |
| Thrombocytopenia (platelet count < 50,000/mm^3^) with history of intravenous platelet transfusion | Objectives 3 and 4 | Allscripts EHR | ICD; CPT | SNOMED CT; Lab | 2 clinician review |
| **Others** |  |  |  |  |  |
| HIV Infection | Observational | Allscripts EHR | ICD | SNOMED CT; Lab | 2 clinician review |
| Known diagnosis of Cancer with the exception of non-melanoma skin cancer | Observational | Allscripts EHR | ICD | SNOMED CT; Lab | 2 clinician review |

Supplementary Table C: The SNOMED-CT code retained for inclusion criteria is given in the table below:

| ***inclusion*** | *snomed_ct_code* | *label* |
| --- | --- | --- |
| Atrial fibrillation | 49436004 | Atrial fibrillation (disorder) |

The SNOMED-CT codes retained for exclusion criteria are detailed in the table below:

| ***exclusion*** | *snomed_ct_code* | *Label* |
| --- | --- | --- |
| Valvular heart disease | 86466006 | Rheumatic mitral stenosis (disorder) |
| Valvular heart disease | 787001 | Rheumatic mitral stenosis with regurgitation (disorder) |
| Valvular heart disease | 31085000 | Rheumatic mitral regurgitation (disorder) |
| Valvular heart disease | 194737007 | Multiple mitral and aortic valve involvement (disorder) |
| Valvular heart disease | 194733006 | Mitral and aortic stenosis (disorder) |
| Valvular heart disease | 194732001 | Diseases of mitral and aortic valves (disorder) |
| Valvular heart disease | 194734000 | Mitral stenosis and aortic insufficiency (disorder) |
| Valvular heart disease | 11851006 | Mitral valve disorder (disorder) |
| Valvular heart disease | 204315000 | Atrial septal defect within oval fossa (disorder) |
| Valvular heart disease | 30288003 | Ventricular septal defect (disorder) |
| Valvular heart disease | 17107009 | Mitral valve prosthesis, device (physical object) |
| Valvular heart disease | 195002007 | Multiple valve disease (disorder) |
| Valvular heart disease | 34068001 | Heart valve replacement (procedure) |
| Valvular heart disease | 161667004 | History of heart valve recipient (situation) |
| Valvular heart disease | 699347000 | Mechanical heart valve replacement (procedure) |
| Valvular heart disease | 307279007 | Prosthetic replacement of heart valve (procedure) |
| Valvular heart disease | 287312007 | Heart valve replacement - graft (procedure) |
| Valvular heart disease | 85830006 | Repair of heart valve (procedure) |
| Congenital heart disease | 268174004 | Bulbus cordis and cardiac septal closure anomalies (disorder) |
| VTE (DVT and PE) | 59282003 | Pulmonary embolism (disorder) |
| VTE (DVT and PE) | 128053003 | Deep venous thrombosis (disorder) |
| VTE (DVT and PE) | 161508001 | History of deep vein thrombosis (situation) |
| VTE (DVT and PE) | 161512007 | History of pulmonary embolus (situation) |
| VTE (DVT and PE) | 438773007 | Recurrent pulmonary embolism (disorder) |
| VTE (DVT and PE) | 56272000 | Postpartum deep phlebothrombosis (disorder) |
| VTE (DVT and PE) | 111458008 | Postpartum venous thrombosis (disorder) |
| Pericarditis | 3238004 | Pericarditis (disorder) |
| Hyperthyroidism and Thyrotoxicity | 34486009 | Hyperthyroidism (disorder) |
| Pregnancy | 77386006 | Patient currently pregnant (finding) |
| Pregnancy | 198609003 | Complication of pregnancy, childbirth and/or the puerperium (disorder) |

The SNOMED-CT codes retained for the criteria of the scores are detailed in the table below:

| *category* | *snomed_ct_code* |
| --- | --- |
| Congestive heart failure | 84114007 |
| Congestive heart failure | 134378009 |
| Congestive heart failure | 85898001 |
| Congestive heart failure | 233928007 |
| Congestive heart failure | 277455002 |
| Congestive heart failure | 90727007 |
| Hypertension | 10725009 |
| Hypertension | 48146000 |
| Hypertension | 59621000 |
| Hypertension | 71701000119105 |
| Hypertension | 71421000119105 |
| Hypertension | 397748008 |
| Hypertension | 706882009 |
| Hypertension | 62275004 |
| Hypertension | 697929007 |
| Hypertension | 23130000 |
| Hypertension | 31992008 |
| Hypertension | 56218007 |
| Hypertension | 270440008 |
| Diabetes | 73211009 |
| Diabetes | 67866001 |
| Diabetes | 443911005 |
| Stroke | 230690007 |
| Stroke | 266257000 |
| Vascular disease | 400047006 |
| Vascular disease | 399957001 |
| Vascular disease | 28960008 |
| Vascular disease | 72092001 |
| Vascular disease | 414545008 |
| Vascular disease | 251019006 |
| Vascular disease | 232717009 |
| Vascular disease | 415070008 |
| Vascular disease | 108979001 |
| Liver disease | 328383001 |
| Renal disease | 236423003 |
| Bleeding tendency | 74474003 |
| Bleeding tendency | 64779008 |
| Bleeding tendency | 28670008 |
| Bleeding tendency | 236002003 |
| Drug or alcohol use | 26416006 |
| Drug or alcohol use | 361055000 |
| Drug or alcohol use | 7200002 |
| Drug or alcohol use | 219006 |

**Supplemental Table D: Exclusion Codes**

| **Exclusion Codes – Valvular Diagnosis** |  |
| --- | --- |
| Disease of mitral valve | 394.xx |
| Disease of aortic valve | 395.xx |
| Disease of mitral and aortic valves | 396.xx |
| Diseases of other endocardial structures | 397.xx |
| Other diseases of endocardium | 424.xx |
| Syphilitic endocarditis, mitral valve | 093.21 |
| Congenital mitral stenosis | 746.5x |
| Congenital mitral insufficiency | 746.6x |
| Heart valve replaced by transplant | V42.2x |
| Heart valve replaced by other means | V43.3x |

**Supplementary Table E: ICD9 codes utilized**

| Entity | ICD9 |  |  |  |  |
| --- | --- | --- | --- | --- | --- |
| Hypertension | 401.XX | 402.xx | 403.xx | 404.xx | 405.xx |
| CHF | 428.XX |  |  |  |  |
| DM | 250.XX |  |  |  |  |
| stroke or TIA | 434.xx | 433.XX | 436.XX |  |  |
| Thromboembolic Disease | 444.9 | 453.9 | 453.4 | 415.1 |  |
| Vascular disease | 414.XX | 410.XX | 411.xx | 413.XX | 440.XX |
|  |  |  |  |  |  |
| Chronic Renal Insufficiency | 585.3 | 585.4 | 585.6 |  |  |
| Liver disease | 571.XX |  |  |  |  |
| Major bleeding or clotting disorder | 459 | 578.XX | 286.4 | 286.XX |  |
| Medication usage predisposing to bleeding | V58.61 | Z79.01 | V58.63 | V58.64 |  |
| Alcoholism | 303.XX |  |  |  |  |
|  |  |  |  |  |  |
|  |  |  |  |  |  |
|  |  |  |  |  |  |
| Supplementary Table F: RxNorm Codes Utilized  Medication Usage predisposing to bleeding using RxNorm | 197374, 198466, 198467, 198471309362, 749196 313406 |  |  |  |  |
|  | 198475, 198477, 199274, 212033 | | | |  |
|  | 243663, 243670, 246460, 252380 | | | |  |
|  | 259081, 308409, 308411, 308414 | | | |  |
|  | 308416, 308417, 318272, 403924 | | |  | |
|  | 432389, 435504, 435521, 747211 | | | |  |
|  | 853499 |  |  |  |  |
