# Supplementary material for: Using Artificial Intelligence With Natural Language Processing to Combine Electronic Health Record’s Structured and Free Text Data to Identify Nonvalvular Atrial Fibrillation to Decrease Strokes and Death: Evaluation and Case-Control Study
Source: J Med Internet Res. 2021 Nov 9;23(11):e28946. doi: 10.2196/28946 (PMC8663460; doi:10.2196/28946)
Supplement: Multimedia Appendix 2 [file jmir_v23i11e28946_app2.docx]

### Multimedia Appendix 2. Financial and Nonfinancial Support

| Author | Financial support | Nonfinancial support |
| --- | --- | --- |
| Peter Elkin | Pfizer, Inc, NLM T15LM012595, UL1TR001412 NIAAA R21AA026954, R33AA026954 | None to report |
| Sarah Mullin | NLM T15LM012595 | None to report |
| Chris Crowner | VA | None to report |
| Sylvester Sakilay | VA | None to report |
| Shyamashree Sinha | NIH T32GM099607 | None to report |
| Sashank Kaushik | None to report | None to report |
| Jane Zhao | None to report | None to report |
| Buer Song | None to report | None to report |
| Edwin Anand | None to report | None to report |
| Gary Brady | Employee of Pfizer | None to report |
| Marcia Wright | Employee of Pfizer | None to report |
| Jack Mardekian | Employee of Pfizer | None to report |
| JoAnn Trainer | Employee of Pfizer | None to report |
| Kim Nolen | Employee of Pfizer | None to report |
| Ross Koppel (University of Pennsylvania and University at Buffalo) | Employee of University at Buffalo and University of Pennsylvania, received funding from Pfizer to assist with writing and editing | None to report |
